# Supplementary material for: Time-series transcriptome analysis identified differentially expressed genes in broiler chicken infected with mixed Eimeria species
Source: Front Genet. 2022 Aug 8;13:886781. doi: 10.3389/fgene.2022.886781 (PMC9393255; doi:10.3389/fgene.2022.886781)
Supplement: Supplementary file 2 [file DataSheet1.ZIP › 7dpi_KEGG.Gsea.1625115961950/KEGG_PANCREATIC_CANCER.html]

Details for gene set KEGG\_PANCREATIC\_CANCER[GSEA]

|  || Dataset | TMM\_7dpi\_gct\_format\_7dpi\_gct\_format.Class\_4dpi.cls #PC\_versus\_NC.Class\_4dpi.cls #PC\_versus\_NC\_repos |
| Phenotype | Class\_4dpi.cls#PC\_versus\_NC\_repos |
| Upregulated in class | 0 |
| GeneSet | KEGG\_PANCREATIC\_CANCER |
| Enrichment Score (ES) | -0.44973642 |
| Normalized Enrichment Score (NES) | -1.5168011 |
| Nominal p-value | 0.010845507 |
| FDR q-value | 0.12154778 |
| FWER p-Value | 0.9434 |
Table: GSEA Results Summary

  

Fig 1: Enrichment plot: KEGG\_PANCREATIC\_CANCER      
 Profile of the Running ES Score & Positions of GeneSet Members on the Rank Ordered List

  

| SYMBOL | TITLE | RANK IN GENE LIST | RANK METRIC SCORE | RUNNING ES | CORE ENRICHMENT || 1 | STAT1 | na | 103 | 1.240 | 0.0295 | No |
| 2 | BCL2L1 | na | 568 | 0.700 | 0.0122 | No |
| 3 | PGF | na | 1105 | 0.483 | -0.0178 | No |
| 4 | TGFA | na | 1901 | 0.287 | -0.0755 | No |
| 5 | TGFB2 | na | 1954 | 0.278 | -0.0713 | No |
| 6 | CASP9 | na | 2022 | 0.268 | -0.0687 | No |
| 7 | RAC1 | na | 2998 | 0.126 | -0.1464 | No |
| 8 | RALA | na | 3386 | 0.075 | -0.1764 | No |
| 9 | ERBB2 | na | 3936 | 0.011 | -0.2220 | No |
| 10 | RALB | na | 4141 | -0.011 | -0.2388 | No |
| 11 | VEGFC | na | 5000 | -0.103 | -0.3074 | No |
| 12 | RAF1 | na | 5142 | -0.116 | -0.3156 | No |
| 13 | MAP2K1 | na | 5188 | -0.120 | -0.3157 | No |
| 14 | TGFB1 | na | 5297 | -0.130 | -0.3207 | No |
| 15 | RALBP1 | na | 5695 | -0.169 | -0.3487 | No |
| 16 | CDC42 | na | 5837 | -0.184 | -0.3549 | No |
| 17 | RAC3 | na | 6076 | -0.210 | -0.3683 | No |
| 18 | TGFBR2 | na | 6133 | -0.218 | -0.3663 | No |
| 19 | RELA | na | 6283 | -0.233 | -0.3716 | No |
| 20 | MAPK8 | na | 6464 | -0.250 | -0.3790 | No |
| 21 | AKT3 | na | 7033 | -0.312 | -0.4170 | No |
| 22 | E2F1 | na | 7190 | -0.328 | -0.4199 | No |
| 23 | KRAS | na | 7206 | -0.330 | -0.4110 | No |
| 24 | TGFBR1 | na | 7506 | -0.362 | -0.4249 | No |
| 25 | MAPK9 | na | 7648 | -0.377 | -0.4252 | No |
| 26 | CDK6 | na | 7686 | -0.382 | -0.4165 | No |
| 27 | RAC2 | na | 7904 | -0.409 | -0.4221 | No |
| 28 | RB1 | na | 8176 | -0.443 | -0.4312 | No |
| 29 | AKT1 | na | 8399 | -0.472 | -0.4352 | Yes |
| 30 | IKBKB | na | 8465 | -0.479 | -0.4260 | Yes |
| 31 | PIK3CG | na | 8478 | -0.481 | -0.4122 | Yes |
| 32 | VEGFA | na | 8816 | -0.526 | -0.4242 | Yes |
| 33 | PLD1 | na | 8979 | -0.546 | -0.4210 | Yes |
| 34 | RAD51 | na | 9288 | -0.597 | -0.4284 | Yes |
| 35 | NFKB1 | na | 9346 | -0.606 | -0.4146 | Yes |
| 36 | VEGFD | na | 9472 | -0.627 | -0.4058 | Yes |
| 37 | CHUK | na | 9554 | -0.639 | -0.3930 | Yes |
| 38 | TGFB3 | na | 9555 | -0.639 | -0.3733 | Yes |
| 39 | PIK3R2 | na | 9597 | -0.645 | -0.3569 | Yes |
| 40 | ARHGEF6 | na | 9972 | -0.706 | -0.3666 | Yes |
| 41 | SMAD3 | na | 10010 | -0.714 | -0.3477 | Yes |
| 42 | E2F3 | na | 10031 | -0.717 | -0.3274 | Yes |
| 43 | MAPK3 | na | 10320 | -0.774 | -0.3277 | Yes |
| 44 | PIK3CB | na | 10335 | -0.777 | -0.3050 | Yes |
| 45 | CCND1 | na | 10432 | -0.796 | -0.2886 | Yes |
| 46 | PIK3CD | na | 10707 | -0.855 | -0.2852 | Yes |
| 47 | PIK3CA | na | 10920 | -0.912 | -0.2749 | Yes |
| 48 | PIK3R5 | na | 10941 | -0.918 | -0.2484 | Yes |
| 49 | PIK3R3 | na | 11045 | -0.949 | -0.2279 | Yes |
| 50 | STAT3 | na | 11052 | -0.950 | -0.1992 | Yes |
| 51 | BRCA2 | na | 11131 | -0.973 | -0.1758 | Yes |
| 52 | JAK1 | na | 11193 | -0.997 | -0.1503 | Yes |
| 53 | CDKN2A | na | 11228 | -1.009 | -0.1221 | Yes |
| 54 | PIK3R1 | na | 11265 | -1.023 | -0.0937 | Yes |
| 55 | EGFR | na | 11415 | -1.085 | -0.0729 | Yes |
| 56 | BRAF | na | 11724 | -1.264 | -0.0598 | Yes |
| 57 | MAPK10 | na | 12008 | -2.724 | 0.0002 | Yes |
Table: GSEA details [plain text format]

  

Fig 2: KEGG\_PANCREATIC\_CANCER      
 Blue-Pink O' Gram in the Space of the Analyzed GeneSet

  

Fig 3: KEGG\_PANCREATIC\_CANCER: Random ES distribution      
 Gene set null distribution of ES for **KEGG\_PANCREATIC\_CANCER**
